# BDMC33, A Curcumin Derivative Suppresses Inflammatory Responses in Macrophage-Like Cellular System: Role of Inhibition in NF-κB and MAPK Signaling Pathways

**DOI:** 10.3390/ijms13032985

**Published:** 2012-03-06

**Authors:** Ka-Heng Lee, Yuh-Lit Chow, Vidyadaran Sharmili, Faridah Abas, Noorjahan Banu Mohamed Alitheen, Khozirah Shaari, Daud Ahmad Israf, Nordin Haji Lajis, Ahmad Syahida

**Affiliations:** 1 Department of Biochemistry, Faculty of Biotechnology and Biomolecular Sciences, Universiti Putra Malaysia, 43400 UPM Serdang, Selangor, Malaysia; E-Mail: heng1011@yahoo.co.uk; 2 Institute of Bioscience, Universiti Putra Malaysia, 43400 UPM Serdang, Selangor, Malaysia; E-Mails: yuhlit@yahoo.co.uk (Y.-L.C.); faridah@food.upm.edu.my (F.A.); khozirah@science.upm.edu.my (K.S.); daud@medic.upm.edu.my (D.A.I.); mdnordin@science.upm.edu.my (N.H.L.); 3 Department of Pathology, Faculty of Medicine and Health Sciences, Universiti Putra Malaysia, 43400 UPM Serdang, Selangor, Malaysia; E-Mail: sharmili@medic.upm.edu.my; 4 Department of Food Science, Faculty of Food Science and Technology, Universiti Putra Malaysia, 43400 UPM Serdang, Selangor, Malaysia; 5 Department of Cell and Molecular Biology, Faculty of Biotechnology and Biomolecular Sciences, Universiti Putra Malaysia, 43400 UPM Serdang, Selangor, Malaysia; E-Mail: noorjahan@biotech.upm.edu.my; 6 Department of Chemistry, Faculty of Sciences, Universiti Putra Malaysia, 43400 UPM Serdang, Selangor, Malaysia; 7 Department of Biomedical Sciences, Faculty of Medicine and Health Sciences, Universiti Putra Malaysia, 43400 UPM Serdang, Selangor, Malaysia

**Keywords:** anti-inflammatory, BV-2, curcumin, MAPK, NF-κB, RAW 264.7

## Abstract

Our preliminary screening has shown that curcumin derivative BDMC33 [2,6-bis(2,5-dimethoxybenzylidene)cyclohexanone] exerted promising nitric oxide inhibitory activity in activated macrophages. However, the molecular basis and mechanism for its pharmacological action is yet to be elucidated. The aim of this study was to investigate the anti-inflammatory properties of BDMC33 and elucidate its underlying mechanism action in macrophage cells. Our current study demonstrated that BDMC33 inhibits the secretion of major pro-inflammatory mediators in stimulated macrophages, and includes NO, TNF-α and IL-1β through interference in both nuclear factor kappaB (NF-κB) and mitogen activator protein kinase (MAPK) signaling cascade in IFN-γ/LPS-stimulated macrophages. Moreover, BDMC33 also interrupted LPS signaling through inhibiting the surface expression of CD-14 accessory molecules. In addition, the inhibitory action of BDMC33 not only restricted the macrophages cell (RAW264.7), but also inhibited the secretion of NO and TNF-α in IFN-γ/LPS-challenged microglial cells (BV-2). The experimental data suggests the inflammatory action of BDMC33 on activated macrophage-like cellular systems, which could be used as a future therapeutic agent in the management of chronic inflammatory diseases.

## 1. Introduction

Inflammation is a beneficial host immune response to tissue injury or infection. However, prolonged or uncontrolled inflammation may lead to potentially damaging consequences and contribute to pathogenesis of several inflammatory disorders and cancers [1]. Macrophages play a key role in inflammatory response and serve as a decisive interface in bridging both innate and adaptive immunity [2,3]. Lipopolysaccharide (LPS) and interferon-γ (IFN-γ) are an excellent combination of stimulus to act synergistically in macrophage activation [4,5]. LPS binds to TLR-4, facilitated by the CD-14 co-receptor, whereas IFN-γ prime the macrophages for more a rapid and heightened response to LPS [6,7]. TLR-4 stimulation, mediated by LPS, recruits the cytoplasmic adaptor protein MyD88, subsequently activating two distinct downstream signaling pathways, the nuclear factor kappaB (NF-κB) pathway and the mitogen activated protein kinase (MAPK) pathway. These signaling pathways are known to stimulate the release of numerous inflammatory mediators such as nitric oxide (NO), cytokines, prostaglandin E_2_ and other eicosanoid mediators to promote inflammatory response [8].

Nitric oxide is a free radical generated by nitric oxide synthase (NOS) and serves as an important mediator in regulating the physiological and pathophysiological mechanism of cardiovascular, nervous and immunological systems. The expression of inducible NOS (iNOS) is responsible for the prolonged production of NO that mediates the bactericidal and tumoricidal functions of activated macrophages [9]. However, excessive production of NO in chronic inflammation has been implicated in the development of autoimmune disorders and cancer [10]. Besides, a number of pro-inflammatory cytokines are released by macrophages during inflammation, including tumor necrosis factor-α (TNF-α), interleukin-1β (IL-1β) and IL-6 [11]. These cytokines play a significant role in the pathogenesis of various inflammatory diseases and serve as potent biomarkers to assess the inflammatory process [12]. As a result, a number of strategies have been designed to block the expression of these pro-inflammatory mediators to enhance disease management, including the use of a specific inhibitor or the antagonizing effect of antibodies.

NF-κB signaling plays a vital role in regulating cellular proliferation, inflammatory response and cell adhesion [13]. The activation of NF-κB has been reported to induce transcription of multiple pro-inflammatory cytokines and pivotal enzymes that are involved in maintaining inflammation such as iNOS, COX-2, TNF-α, IL-1β, IL-6 and others [14]. Generally, functionally active NF-κB consisting of Rel family p50 and p65 subunits are normally confined to an inactive cytoplasmic complex through binding to an inhibitory protein (I-κB) in unstimulated conditions. The activation of NF-κB causes rapid phosphorylation of I-κB at two critical serine residues (Ser^32^ and Ser^36^) by I-κB kinase (IKK) and the phosphorylated I-κB are targeted for ubiquitination and followed by 26s proteosomal degradation. The dissociation of I-κB enables the free NF-κB to translocate into the nucleus via specific machinery and bind to the κB binding sites in the promoter of the targets genes [15–17]. On the other hand, MAPK is one of the signal transduction cascades involved in the regulation of inflammatory mediator expression in activated macrophages. The MAPK family consists of three main sub-family members, including extracellular signal-regulated kinase (ERK), p38 MAPK, and c-Jun NH_2_-terminal kinase (JNK) [18,19]. Persistent activation of MAPK signaling pathways has been implicated in the development of many human diseases, such as Alzheimer’s disease, Parkinson’s disease, amyotrophic lateral sclerosis and various types of cancers [20]. Therefore, both NF-κB and MAPK signaling pathways serve as important molecular targets for the development of potential anti-inflammatory drugs and present a promising chemotherapeutic strategy to control inflammatory disease.

**Figure 1:**
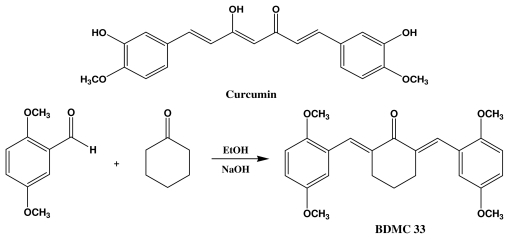
Chemical structure of curcumin (**a**) and synthesis of BDMC33 (**b**).

Curcumin or [1,7-bis-(hydroxy-3-methoxyphenyl)-1,6-heptadiena-3,5-dione] is a yellow pigment and naturally occurring phytochemical found in the rhizome of *Curcuma longa* L. (commonly known as turmeric) (Figure 1a). Within the last decade, the therapeutic and chemoprevention properties of curcumin have been extensively studied because of its wide spectrum of pharmacological activity, such as antioxidant, anti-proliferative, anti-carcinogenic, anti-angiogenic, anti-bacterial, immune-modulatory, and anti-inflammatory [21]. Pre-clinical studies have shown that curcumin is a highly pleiotophic molecule with immunomodulatory effects on different cellular models in preventing various inflammatory disorders, such as rheumatoid arthritis, neurodegeneration, inflammatory bowel disease and cardiovascular disease [22]. Curcumin has appeared to be a promising chemopreventive compound, which was proved to be safe and effective in many clinical trials. However, its clinical advancement has been hampered due to its poor pharmacokinetic properties [23]. It was believed that the instability of the curcumin structure was contributed to by its active methylene group and the β-diketone moiety, which renders curcumin to be easily degraded by aldo-keto reductase in the liver [24]. Our group has adopted the chemical synthesis of a curcumin analogue by eliminating the unstable methylene group and β-diketone moiety, to overcome the limitation on its bioavailability. We previously have reported that BDMC33 [2,6-bis(2,5-dimethoxybenzylidene) cyclohexanone] exhibited improved anti-inflammatory activities by inhibiting NO production in the IFN-γ/LPS-challenged macrophages cell (RAW 264.7) [25]. However, the cellular and molecular mechanism underlying BDMC33-mediated inhibition of NO production in macrophages has yet to be elucidated. The present study provides evidence that BDMC33 exhibited its anti-inflammatory activity via suppression of NF-κB activation and AP-1 activities by blockade of ERK/JNK signaling pathways.

## 2. Results

### 2.1. Inhibitory Action on NO Production via Down-Regulation of iNOS Expression

**Figure 2:**
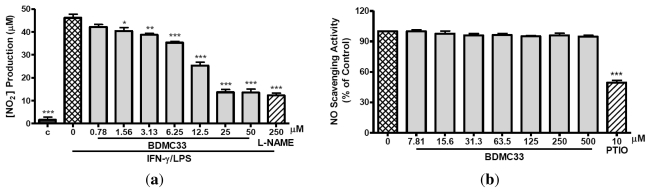

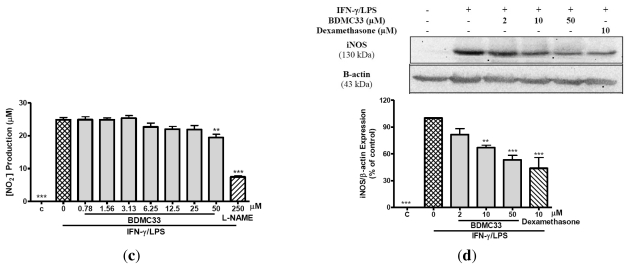
Effects of BDMC33 on NO production, NO scavenging activity (cell-free system), iNOS activity and iNOS expression in IFN-γ/LPS-induced RAW 264.7 macrophages. (**a**) Cells were stimulated for 17–20 h with 100 U/mL recombinant murine IFN-γ and 5 μg/mL *E. coli* LPS and treated with increasing concentrations of BDMC33. The IC_50_ was calculated at 13.66 ± 0.61 μM. Nitrite level was determined by the Griess reaction after treatment. L-NAME (250 μM) was used as standard iNOS inhibitor for NO inhibition; (**b**) Percentage of nitrite accumulation produced by sodium nitropruside (SNP) in the presence or absence of BDMC33 was determined by Griess assay. PTIO was used as positive control as a NO scavenger; (**c**) Cells were treated with IFN-γ/LPS for 12 h prior to treatment with increasing concentrations of BDMC33. L-NAME (250 μM) was used as a standard iNOS inhibitor for NO inhibition. Nitrite level was determined by Griess reaction after treatment; (**d**) Cells were stimulated for 20 h with combination of IFN-γ/LPS and treated with increasing concentrations of BDMC33. Whole cell lysates were assayed for iNOS expression by using Western blotting. Immunoblotting of β-actin expression was used as loading control. Dexamethasone (DXM) was used as positive control for iNOS expression. The expression level was normalized against IFN-γ/LPS treated group. C, Basal level of nitrite/iNOS expression without IFN-γ/LPS treatment. All values are expressed in mean ± standard error of mean (S.E.M.) of three independent experiments. * *P* < 0.05, ** *P* < 0.01, *** *P* < 0.001 were significantly different from the IFN-γ/LPS-treated control group (second column).

The induction of RAW 264.7 cells into an inflammatory state by combination treatment of IFN-γ/LPS results in synthesis and secretion of NO. In Figure 2a, BDMC33 shows dose-related inhibition of NO production in which significant inhibition was still evident at 1.56 μM (*P* < 0.05) and the IC_50_ was calculated at 13.66 ± 0.61 μM. L-NAME, a standard NOS inhibitor, was used as positive drug control and significantly inhibited NO production (73.45 ± 1.94%) at 250 μM. The question is whether the inhibition of NO secretion was due to the BDMC33 effect on intracellular targets or just the scavenging of secreted NO. Figure 2b shows that BDMC33 did not scavenge NO free radicals at all concentrations tested. Then, we examined whether the inhibitory action of BDMC33 on NO production was due to the suppression of iNOS activity or its expression. As demonstrated in Figure 2c, BDMC33 showed a slight reduction in nitrite synthesis at a concentration of 50 μM and had minimal inhibitory effect upon iNOS activity. However, western blotting analysis demonstrated BDMC33 showed a significant dose-dependent, down-regulatory effect upon iNOS protein expression; doses as low as 10 μM were significantly suppressive (*P* < 0.01). Dexamethasone, a potential anti-inflammatory steroid hormone, also significantly inhibited iNOS expression (43.88 ± 11.97%) at a concentration of 10 μM (Figure 2d). In general, these results indicated that the inhibitory action of BDMC33 on IFN-γ/LPS-induced NO production mainly results from the suppression of iNOS protein.

### 2.2. Interference in Pro-Inflammatory Cytokine Production

**Figure 3:**
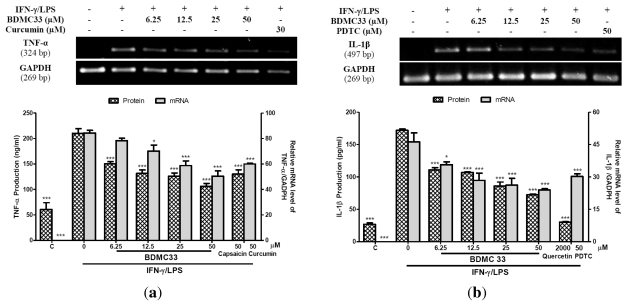
(**a**) Effect of BDMC33 on TNF-α production and gene expression in IFN-γ/LPS-induced RAW 264.7. The IC_50_ was calculated at 9.40 ± 1.67 μM. Capsaisin (50 μM) and curcumin (30 μM) were used as positive drug control for protein and mRNA expression level of TNF-α respectively; (**b**) Effect of BDMC33 on IL-1β production and gene expression in IFN-γ/LPS-induced RAW 264.7. The IC_50_ was calculated at 29.66 ± 0.72 μM. Quercetin (2000 μM) and pyrroline dithiocarbamate (PDTC) (30 μM) were used as positive drug control for protein and mRNA expression level of IL-1β respectively. C; Basal protein and mRNA expression level of TNF-α/IL-1β without IFN-γ/LPS treatment. All values are expressed in mean ± S.E.M. of three independent experiments. * *P* < 0.05; *** *P* < 0.001, significantly different from IFN-γ/LPS-treated control group.

Tumor necrosis factor-α and IL-1β are pleiotropic cytokines that have been implicated as key pro-inflammatory cytokines in immune and inflammatory response. Thus, we investigated the effects of BDMC33 on cytokine production and its gene expression by ELISA and semi-quantitative RT-PCR respectively. Figure 3a showed the effects of various treatments on TNF-α production and its gene expression in IFN-γ/LPS-induced RAW 264.7 cells. BDMC33 demonstrated a dose-dependent inhibitory effect upon TNF-α production in which doses as low as 6.25 μM were significantly suppressive (*P* < 0.001) and the IC_50_ was calculated at 9.40 ± 1.67 μM. Capsaicin was used as positive drugs control and significantly inhibited the production of TNF-α (53.86 ± 2.77%). Semi-quantitative RT-PCR results revealed that IFN-γ/LPS treatment to RAW 264.7 cells caused the augmentation of *TNF-α* gene expression. In addition, BDMC33 displayed a dose-dependent inhibition in *TNF-α* gene expression as low as 12.5 μM (*P* < 0.05). Curcumin (30 μM) was used as positive control and showed slight inhibition (38.77 ± 2.01%) of *TNF-α* gene expression but to a lesser degree than BDMC33.

Our results demonstrated that BDMC33 also suppressed IL-1β synthesis in a similar trend as observed for TNF-α synthesis. Figure 3b showed the effects of various treatments on protein and mRNA level of IL-1β in RAW 264.7 cells. BDMC33 inhibited IL-1β production in a dose-dependent manner in which as little as 6.25 μM was significantly inhibited (*P* < 0.001) and the IC_50_ was calculated at 29.66 ± 0.72 μM. Quercetin was used as a positive drugs control and strongly inhibited the production of IL-1β (90.09 ± 2.88%). Furthermore, the gene expression of *IL-1β* was found to be significantly inhibited by BDMC33 in a dose-dependent manner. PDTC (50 μM) was used as a positive control and showed significant inhibition (43.69 ± 4.66%) of *IL-1β* gene expression but to a lesser degree than BDMC33.

### 2.3. Attenuation of NF-κB and AP-1 DNA Binding Activity

**Figure 4:**
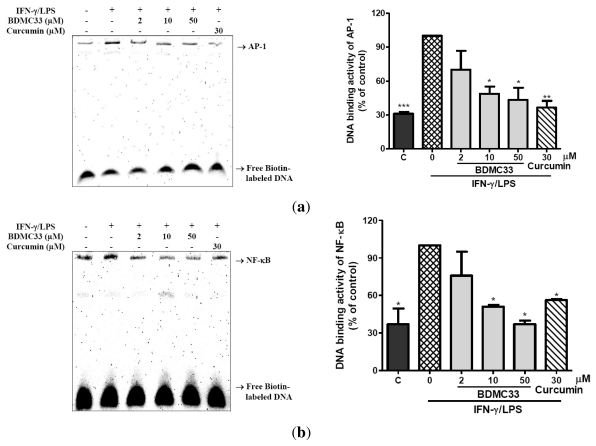
Effect of BDMC33 on DNA binding activity of transcriptional factors in IFN-γ/LPS-induced RAW 264.7 macrophages. Cells were stimulated with IFN-γ/LPS and treated with increasing concentrations of BDMC33 for 2 h. The nuclear protein was extracted and tested for DNA binding of (**a**) AP-1 and (**b**) NF-κB by EMSA. Curcumin (30 μM) was used as positive drug control for DNA binding activity of both NF-κB and AP-1 transcriptional factors. The expression level was normalized against the IFN-γ/LPS treated group. C, Basal DNA binding activity of NF-κB and AP-1 without IFN-γ/LPS treatment. The results are expressed in mean ± S.E.M. of three independent experiments. * *P* < 0.05, ** *P* < 0.01, *** *P* < 0.001 are significantly different from the IFN-γ/LPS -treated control group (second column).

To further elucidate the underlying molecular mechanism mediated by BDMC33, the DNA binding activity of two important transcriptional factors (NF-κB and AP-1) involved in pro-inflammatory gene expression regulation were examined. Stimulation of LPS/IFN-γ resulted in a significant increase in DNA binding activity of both NF-κB and AP-1, as evaluated by electrophoretic mobility shift assay (EMSA). The specificity of binding was examined by competition with the unlabeled oligonucleotide (data not shown). We have demonstrated that the DNA binding activity of both NF-κB and AP-1 were significantly reduced in a nuclear extract obtained from IFN-γ/LPS-induced RAW 264.7 concomitant treated with different concentrations of BDMC33 (Figure 4). Curcumin was used as a positive control and showed significant inhibition in DNA binding activity of AP-1 (63.54 ± 6.12%) and NF-κB (43.82 ± 0.74%).

### 2.4. Effects of BDMC33 on NF-κB Activation

**Figure 5:**
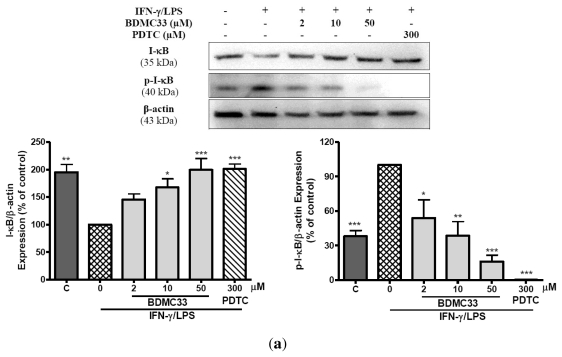

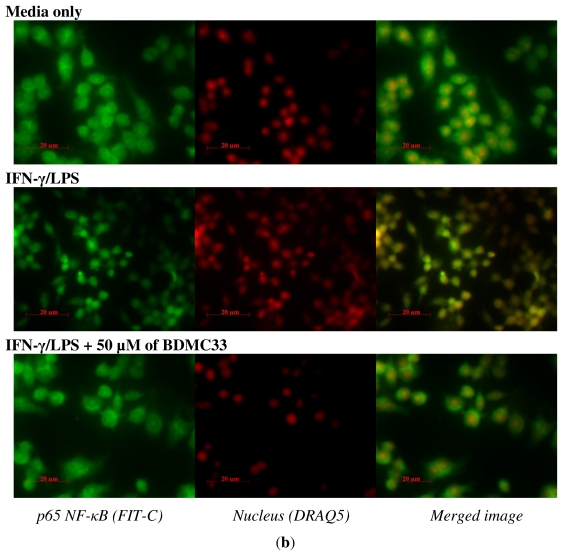
Effects of BDMC33 on I-κB degradation and I-κB phosphorylation as well as NF-κB nuclear translocation in IFN-γ/LPS induced RAW 264.7 macrophages. (**a**) Cell lysate analyzed by western blotting using anti-phospho-I-κB and anti-I-κB antibodies. Expression of β-actin expression was used as loading control and normalized against IFN-γ/LPS treated group; (**b**) Cells cultured in chamber slide were stimulated with IFN-γ/LPS and treated with increasing concentrations of BDMC33. The cellular localization of p65 NF-κB (fluorescein isothiocynate (FTIC) stained, green fluorescent) and nucleus region (DRAQ5 stained, red fluorescent) of cells were identified by immunofluorescence microscopy. All values are the mean ± S.E.M. of three independent experiments. * *P* < 0.05, ** *P* < 0.01, *** *P* < 0.001 are significantly different from IFN-γ/LPS-treated control group.

To gain further insight into the mechanism of BDMC33-mediated regulation of NF-κB activation, we examined the inhibitory effects of BDMC33 on degradation and phosphorylation of I-κB as well as nuclear translocation of the NF-κB p65 subunit. In cells stimulated with IFN-γ/LPS, the expression level of the I-κB was down-regulated, concomitant with the increase of phosphorylated I-κB protein. However, the treatment of BDMC33 prominently sustained I-κB expression levels and inhibited the expression of phosphorylated I-κB protein in a concentration-dependent manner. PDTC, a standard NF-κB inhibitor, was used as positive drug control to maintain the I-κB protein level and showed strong inhibition in phosphorylated I-κB expression (Figure 5a). The IFN-γ/LPS-induced degradation of I-κB can cause the release of NF-κB from the cytoplasmic NF-κB/I-κB complex and allow its translocation into the nucleus of cells. Therefore, we further investigate whether BDMC33 prevents the translocation of the p65 NF-κB into the nucleus after release from the I-κB. As shown in Figure 5b, the p65 subunit mainly localized on the cytoplasm region of the non-induced macrophages and stimulation of cells with IFN-γ/LPS resulting in p65 accumulation in cell nuclei. We have demonstrated that the treatment of BDMC33 (50 μM) abrogated the IFN-γ/LPS-induced p65 nuclear translocation.

### 2.5. Effects of BDMC33 on MAPKs Phosphorylation

**Figure 6:**
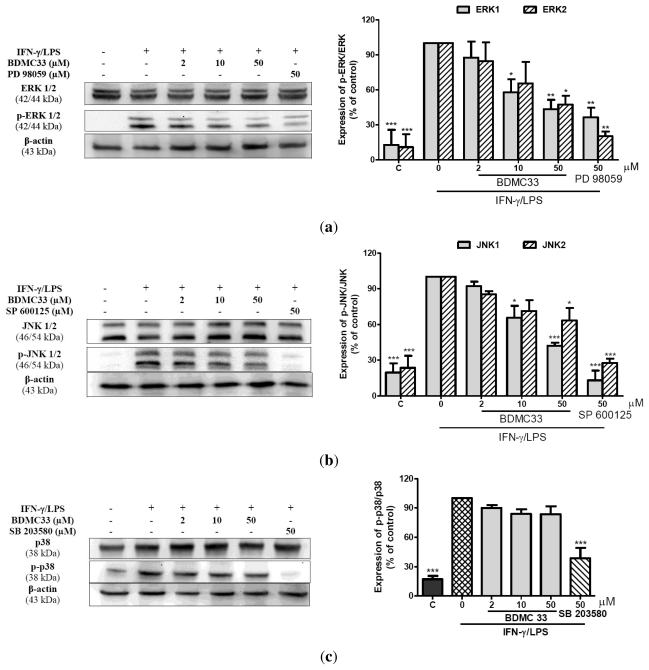
Effects of BDMC33 on MAPKs activation in IFN-γ/LPS-induced RAW 264.7 macrophages. Cells were stimulated with IFN-γ/LPS and treated with increasing concentrations of BDMC33 for 30 min. Whole cell lysates were analyzed with western blotting with specific antibodies. PD98059, SP600125 and SB203580 were used as standard inhibitors for (**a**) ERK, (**b**) JNK and (**c**) p38 activation, respectively. The ratio of immunointensity between the phospho-MAPKs (p-ERK1/2, p-JNK1/2 and p-p38) and total-MAPKs (ERK1/2, JNK1/2 and p38) were calculated from three independent experiments. Expression of β-actin expression was used as loading control and normalized against IFN-γ/LPS treated group. C; Basal level of MAPK expression without IFN-γ/LPS treatment. The results are expressed in mean ± S.E.M. of three independent experiments. * *P* < 0.05, ** *P* < 0.01, *** *P* < 0.001, significantly different from IFN-γ/LPS-treated control group (second column).

To investigate whether the ERK, JNK and p38 MAPK (mitogen-activated protein kinase) are involved in suppression of inflammatory response by BDMC33, we next examined the activation of three subfamilies of MAPKs in detecting their phosphorylated forms by using Western blotting probed with specific anti-phosphokinase antibodies. The exposure of macrophages to IFN-γ/LPS resulted in a strong transient increase of phosphorylated ERK1/2, JNK1/2 and p38 levels at 30 min. As shown in Figure 6, BDMC33 suppressed IFN-γ/LPS–induced activation of ERK1/2 and JNK 1/2 in a concentration-dependent manner, concomitantly, the amount of non-phosphorylated ERK1/2 and JNK1/2 were unaffected either by IFN-γ/LPS or BDMC33 treatment. Although BDMC33 showed minor inhibition of p38 MAPK phosphorylation, but the change was not statistically significant. Standard positive drug controls (PD 98059, SP 600125 and SB 203580) were used and showed strong inhibition of the ERK1/2, JNK1/2 and p38 MAPK activation respectively.

### 2.7. Effects of BDMC33 on CD-14 and TLR-4 Expression

**Figure 7:**
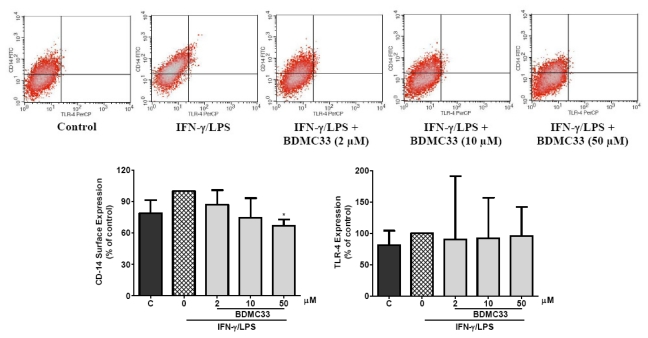
Effects of BDMC33 on IFN-γ/LPS-treated surface expression of TLR-4 and CD-14 accessory molecules in RAW 264.7 macrophages. Cells were stimulated with IFN-γ/LPS and treated with increasing concentrations of BDMC33 for 16 h. The cells were then collected and stained with FITC-conjugated anti-mouse CD-14 and PerCP-conjugated anti-mouse TLR-4 and acquired on FACSCalibur flow cytometer. The expression level was normalized against a IFN-γ/LPS-treated group. C; Basal level of CD-14 or TLR-4 without IFN-γ/LPS treatment. The results are expressed in mean ± S.E.M. of three independent experiments. * *P* < 0.05, significantly different from IFN-γ/LPS-treated control group (second column).

The level of accessory molecule expression on the surface of macrophage cells reflects their inflammatory responsiveness. To investigate whether BDMC33 modulates the accessory molecules on activated macrophages, the surface expression of TLR-4 and CD-14 of macrophages was analyzed by flow cytometer. As shown in Figure 7, the CD-14, constitutively expressed in a relatively high level and exposure of IFN-γ/LPS, caused the slight augmentation of CD-14 expression in RAW 264.7 cells, but was not statistically significant. Conversely, the expression of TLR-4 was relatively low and remained unchanged following IFN-γ/LPS treatment. The treatment of BDMC33 significantly inhibited the expression of CD-14 by approximately 32.30 ± 3.45% at the highest concentration tested (50 μM), while the TLR-4 expression was not altered by BDMC33 treatment.

### 2.8. Effects of BDMC33 on NO and TNF-α Production from Microglial BV-2 Cells

**Figure 8:**
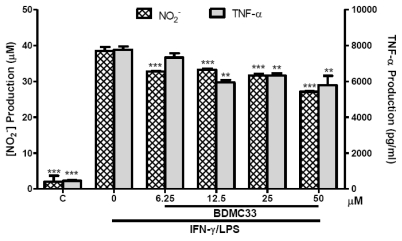
Effects of BDMC33 on NO and TNF-α production in IFN-γ/LPS-induced BV-2 microglia cells. Cells were stimulated with IFN-γ/LPS and treated with increasing concentrations of BDMC33. Concentrations of NO_2_^−^ and TNF-α in the media was determined by Griess assay and ELISA, respectively. C, Basal level of NO_2_^−^/TNF-α without IFN-γ/LPS treatment. All values are the mean ± S.E.M. of three independent experiments. * *P* < 0.05, ** *P* < 0.01, *** *P* < 0.001, significantly different from IFN-γ/LPS-treated control group.

**Figure 9:**
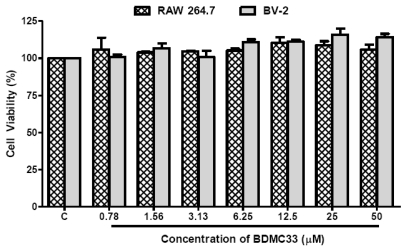
Effects of BDMC33 on cell viability of RAW 264.7 and BV-2 cells. The cells were seeded into a 96-well plate were treated with increasing concentrations of BDMC33 for 24 h and the cell viability determined by MTT assay. C, Basal level of cell viability without IFN-γ/LPS treatment. All values are mean ± S.E.M. of three different independent experiments.

We next addressed the question of whether the inhibitory action of BDMC33 on inflammatory mediator production was restricted to RAW 264.7 macrophages, so the experiments were repeated by replacement of the RAW264.7 cells with BV-2 microgial cells. Stimulation with IFN-γ/LPS effectively augmented the NO (38.47 ± 1.08 μM) and TNF-α (7744.6 ± 193.47 pg/mL) production in the culture medium of BV-2 microglial cells (Figure 8). The production of NO and TNF-α from IFN-γ/LPS-induced microglial cells were found significantly suppressed by BDMC33, albeit in the low level as compared in RAW 264.7 culture system. This NO inhibitory action was not due to cytotoxicity by BDMC33 as assessed in MTT assay (Figure 9).

## 3. Discussion

It is well known that excessive production of NO is harmful. Pharmacological interference in the NO production cascade served as a promising chemotherapeutic strategy in anti-inflammatory agent development. In the present study, we have demonstrated a novel synthetic compound, BDMC33, which inhibited IFN-γ/LPS-induced NO production in RAW 264.7 macrophages *in vitro*. The NO inhibitory action of BDMC33 was neither mediated by its radical scavenging properties nor did it inhibit iNOS enzyme activity, but rather through the down-regulation of iNOS expression. The dose-dependent inhibition behavior of BDMC33 on NO production via iNOS expression showed that the blockage was at the transcriptional regulation level or up-stream signaling molecule. Several studies have also reported that curcumin and its derivatives suppressed iNOS expression in macrophages via interference of the up-stream signaling pathways, especially on NF-κB and MAPK activation [26–28].

Interleukin-1β and TNF-α are pro-inflammatory cytokines that exert pleiotrophic effects on a variety of cells and play a crucial role in acute and chronic inflammatory as well as autoimmune disorders. Both cytokines have been implicated in the pathogenesis of the experimental joint inflammation model, whereby administration of the antagonizing antibodies are effective in prevention of disease progression and joint damage [29,30]. Data presented herein showed that BDMC33 treatment possessed a significant suppressive effect on TNF-α and IL-1β secretion in activated macrophages. The RT-PCR results demonstrated that the mRNA expression of TNF-α and IL-1β were also dose-dependently suppressed by BDMC33 treatment, again suggesting interference at the transcriptional level of the cognate gene by BDMC33 treatment. Interestingly, Moon *et al.* (2010) have reported that curcumin effectively attenuated arthritic disease progression via suppression of TNF-α and IL-1β production in the collagen-induced arthritis (CIA) mouse model [31]; therefore, it is not surprising that BDMC33 might have an anti-arthritic effect *in vivo*.

The regulation of inflammatory mediators is known to occur predominantly at the transcription level, whereby the transcriptional output is tightly controlled by a variety of transcriptional factors, including NF-κB, AP-1, interferon regulator factor 1 (IRF-1), CCAAT/enhancer-binding protein β (C/EBPβ) and cAMP response element binding (CREB) (Adcock & Caramori, 2001). Among these transcriptional factors, NF-κB and AP-1 are the key regulators for the transcription of *iNOS*, *TNF-α* and *IL-1β* [32–34]. The EMSA results demonstrated that the IFN-γ/LPS-induced DNA binding activities of AP-1 and NF-κB were markedly inhibited by BDMC33 in a dose-dependent manner. In addition, curcumin has been shown to inhibit transcriptional activity of NF-κB and AP-1 in primary microglial cells and human monocytes [35,36]. Based on these observations, we believed that the ability of BDMC33 to retain the basal biological activity of curcumin and its suppressive effect on inflammatory genes expression were mediated by the DNA binding activities of AP-1 and NF-κB. However, we cannot exclude the inhibition role of other transcriptional factors, since it has been proposed that IRF-1 and C/EBPβ are also essential factors in *iNOS* activation [37,38].

NF-κB is known to play a crucial role in inflammatory gene regulation, whereby activation of NF-κB results in phosphorylation, ubiquitinition and proteasome-mediated degradation of I-κB protein, followed by nuclear translocation and DNA binding activity of NF-κB. We demonstrated that BDMC33 treatment significantly interrupted NF-κB activation by preventing the degradation of I-κB and suppressed the formation of phosphorylated I-κB, thus resulting in attenuation of p65 NF-κB nuclear translocation and affecting its DNA binding activity. These results suggested that BDMC33 plays a possible role of inhibition on the up-stream protein of I-κB such as IKK or 26s proteosome. Previously, arsenite, cyclopentenone prostaglandin, s-nitrosothiol and anti-rheumatic gold compound have been reported to cause covalent modification of the IKKβ subunit by targeting a critical Cys^179^ residue, resulting in failure of NF-κB activation [39–42]. Interestingly, Conroy & Seto (1998) reported that ketone moiety of cyclohexanone-based inhibitors was able to form a reversible covalent hemithioketal bond with active site cysteine residue of papain, a cysteine protease [43]. Therefore, the ketone moiety present at the cyclohexanone skeleton of BDMC33 might be essential for the covalent modification of the IKKβ subunit via interaction with the Cys^179^ residue, resulting in attenuation of NF-κB activation.

MAPK is an important kinase-mediated signaling pathway in regulating inflammatory gene expression of cells in response to an extraordinarily diverse array of stimuli such as cytokine and endotoxin. In this study, we found that BDMC33 significantly inhibited the phosphorylation of ERK and JNK, but not p38 MAPK, in IFN-γ/LPS-induced macrophage. It was believed that the attenuation of AP-1 DNA binding activities by BDMC33 was mediated via the suppression of ERK1/2 and JNK1/2 phosphorylation. MAPK/ERK kinase kinase (MEKK) 1–4, are the up-stream serine/threonine protein kinases of MAPKs which have been shown to cause strong stimulation of ERK and JNK activities, but inefficiently activate the p38 MAPK activity in human embryonic kidney cell (HEK 293) [44]. In addition, MEKK 2–3 stimulate the activation of NF-κB pathways through IKK-α and IKK-β activities in *in vivo* experiments [45]. Interestingly, Xue *et al.* (2005) reported that the electrophilic ketone moiety of the cyclohexanone-based inhibitor suppressed proteolytic activity of plasmin via interaction with the serine residue of the enzyme to give a reversibly formed hemiketal linkage [46]. Base on these observations, we suggested that the inactivation of JNK and ERK, as well as the NF-κB pathway by BDMC33, might result from the inhibition of the MEKKs’ activities by targeting the active site serine residue of the MEKKs. Furthermore, Palusiaka and Grabowski (2002) reported that the methoxy- group was a proton acceptor in hydrogen bonding. Thus, we believed that the methoxy-substitution group in the phenyl ring of BDMC33 was an excellent proton acceptor to form a hydrogen bond with the active site residue of the up-stream signaling molecules [47]. This was further supported by Pae *et al.* (2008) who suggested that the methoxy- group of the dimethoxycurcumin (a curcumin derivative) was a significant factor in inhibiting NO production, iNOS expression and NF-κB activation upon activated macrophages [48].

Accessory molecules such as TLR-4 and CD-14 play a critical role in LPS-mediated stimulation of macrophages [6]. In our present study, BDMC33 was shown to inhibit the surface expression of co-receptor CD-14, but not TLR-4. Recently, inhibition of CD-14 has been shown effectively to attenuate early inflammatory response in the animal model of sepsis [49]. In addition, IC14, a recombinant chimeric monoclonal antibody recognizing human CD-14 has shown the prominent suppressive effects on endotoxin-mediated inflammatory responses by antagonizing the inflammatory action of CD-14, and suppressed the TNF-α production *in vivo* and *ex vivo* [50,51]. Therefore, we postulated that the inhibition of the TNF-α and IL-1β production by BDMC33 were partly mediated by inhibition of the CD-14 surface expression, and subsequent reduced the LPS responsiveness to macrophage activation.

Microglial cells are macrophage-like cells that reside in the central nervous system and are crucial in the brain tissue defense system. However, persistent activation of microglial cells is believed to contribute to neurodegenerative disease such as Alzheimer’s disease, Parkinson’s disease and amyotrophic lateral sclerosis; through increasing the release of pro-inflammatory mediators or cytokines, including NO, TNF-α and ROS [52]. With an interest in determining possible cell-type specific effects of BDMC33, we showed that BDMC33 exhibits inhibitory activities on the production of NO and TNF-α in activated microglial cells, albeit at a low inhibition level. This indicates that the suppressive effects were not restricted to RAW 264.7 macrophages. Several studies have shown that LPS and glycosaminoglycan were less capable of activating the ERK signaling pathway in BV-2 cells, suggesting that insensitivity of ERK activation in BV-2 cells by extracellular stimulus [53,54]. Therefore, it was speculated that the immunomodulatory effect of BDMC33, observed in the BV-2 cellular system, is more likely to act strongly on the ERK signaling pathway due to the low suppressive effect.

## 4. Experimental

### 4.1. Materials

The following reagents were obtained commercially: Antibiotic (5000 U/mL penicillin and 5000 μg/mL streptomycin), Dulbecco’s Modified Eagle’s Medium (DMEM) from Flowlab™ (North Ride, Australia); Fetal bovine serum from iDNA Biotechnology Bte Ltd (Singapore); recombinant mouse IFN-γ from eBioscience Inc. (San Diego, CA, USA); *Escherichia coli* (strain 055:B5); 3-(4,5-dimethylthiazol-2-yl)-2,5-diphenyl tetratzolium bromide (MTT) from Fluka Chemie GmbH (Buchs, Switzerland); dimethyl sulfoxide (DMSO), sulphanilamide, naphthylenediamine, Nω-nitro-l-arginine-methyl ester hydrochloride (L-NAME), 2-phenyl-4,4,5,5-tetramethyllimidazoline-1-oxy-2-oxide (PTIO) from Sigma Chemical Co. (St. Louis, MO, USA). Rabbit polyclonal against mouse iNOS antibodies from Cayman Chemicals (Ann Arbor, M, USA). Rabbit polyclonal against human p65, ERK1/2, pERK1/2, p38, pp38, I-κB, pI-κB, HRP conjugated anti-β-actin, fluorescein isothiocynate (FIT-C) conjugated anti-mouse TLR-4 and Peridinin chlorophyll protein complex (PerCP) conjugated anti-mouse CD-14 from Santa Cruz Biotechnology (Santa Cruz, CA, USA). Rabbit polyclonal against human JNK1/2, pJNK1/2 were purchased from Cell Signaling Inc. (Danvers, MA, USA). One-step RT-PCR kit and RNeasy Extraction kit were purchase from Qiagen (Valencia, CA, USA). The polyvinylidene fluoride (PVDF) membrane, HRP conjugated donkey anti-rabbit IgG and Enhanced Chemiluminescence Western Blotting Reagent (ECL) were purchased from Amersham Bioscience UK Ltd. (Buckinghamshire, UK). Nuc-Buster protein extraction kit from Novagen Inc. (Madison, WI, USA). LightShift Chemiluminescent EMSA Kit, Biodyne B positively charged nylon membrane and Biotin labeling kit from Pierce Biotechnology Inc. (Rockford, IL, USA). Gene and protein ladder from Fermentas (Glen Burnie, MD, USA).

### 4.2. Synthesis of 2,6-Bis(2,5-dimethoxybenzylidene)Cyclohexanone (BDMC33)

BDMC33 or 2,6-bis(2,5-dimethoxybenzylidene)cyclohexanone was chemically synthesized (Figure 1b) at the Institute of Bioscience, Universiti Putra Malaysia as describe previously [25].

### 4.3. Cell Culture

The murine macrophage-like cell line (RAW 264.7) from European Collection of Cell Cultures (Porton Down, UK) and murine microglial cell line (BV-2), (a kind gift from Sharmili Vidyadaran, UPM), were both maintained in DMEM supplemented with 10% FBS, 4.5 g/L glucose, sodium pyruvate (1 mM), L-glutamine (2 mM), streptomycin (50 μg/mL) and penicillin (50 U/mL) at 37 °C and 5% CO_2_. When RAW 264.7 and BV-2 cells reached confluency of 80–90%, the cells were scraped out and trypsinized, respectively followed by centrifugation at 110 *xg* at 4 °C for 10 min. The cell viability of cultured cells used in the assay was always >95%, as determined by trypan blue dye exclusion.

### 4.4. Cell Viability

The cytotoxicity of the BDMC33 on cultured cells was determined by assaying the reduction of MTT reagents to formazan salts. After treatment, the supernatant of 96-wells plate containing cells were removed and MTT reagents (0.05 mg/mL) were added into each well. The cells were incubated in 37 °C for 4 h and the formazan salts were dissolved by adding 100% DMSO. The absorbance was then measured at 570 nm on a SpectraMax Plus Microplate reader (Molecular Devices Inc., Sunnyvale, CA, USA).

### 4.5. Measurement of Nitric Oxide Production

RAW 264.7 or BV-2 cells (4 × 10^5^ cells/well) were seeded into a tissue culture grade 96-well plate except for blank and incubated for 2 h at 37 °C, 5% CO_2_ for cell attachment. The attached cells were activated with 100 U/mL of recombinant IFN-γ and 5 μg/mL of LPS with or without the presence of BDMC33 at a final volume 100 μL/well. DMSO was used as vehicle to add BDMC33 to the culture medium and the final concentration of DMSO was 0.1% in all cultures. Cells were then incubated at 37 °C, 5% CO_2_ for 17–20 h. The production of NO was determined by measuring the accumulation of nitrite, which was the stable metabolite of NO in culture medium by using Griess Assay. Briefly, an equal volume of Griess reagent (1% sulphanilamide and 0.1% N-(1-naphty)ethylenediamine in 2.5% H_3_PO_4_), mixed with culture supernatant and color development, was quantified at 550 nm with a SpextraMax Plus microplate reader (Molecular Device). The amount of nitrite in the samples was calculated from a standard curve (0–100 μM) of a freshly prepared sodium nitrite.

### 4.6. Nitrite-Scavenging Activity

Briefly, 50 μL of serially-diluted BDMC33 were added into a 96-well plate. Then, 50 μL of sodium nitroprusside (10 mM dissolved in PBS) solution was added into each well and incubated for 60 min with light exposure. The nitrite levels of the mixture were then determined by Griess assay as described in Section 2.5.

### 4.7. Indirect Determination of iNOS Activity

RAW 264.7 cells (4 × 10^5^ cells/well) were seeded into a tissue culture grade 96-well and triggered with IFN-γ/LPS for 12 h. Then, the cells were washed three times with phosphate buffer saline (PBS) and treated with increasing concentrations of BDMC33 for a further 12 h. The nitrite concentration of supernatant was determined by Griess assay as described in Section 2.5.

### 4.8. Cytokine Immunoassay

The cell culture supernatants were collected and analyzed for TNF-α and IL-1β secretion using commercial ELISA kits (eBioscience, USA). The protocols provided by the manufacturers were followed to the detail. The data was acquired using a SpextraMax Plus microplate reader (Molecular Device). The concentration of TNF-α and IL-1β for each sample was calculated from their respective standard curves.

### 4.9. Semi-Quantitative RT-PCR

RAW 264.7 cells were triggered with IFN-γ/LPS as described earlier and treated with increasing concentrations of BDMC33 for 6 h. The total RNA from RAW 264.7 cells was extracted by using RNeasy Mini extraction kit (Qiagen, USA) and the primer sequences of the target genes were designed by using FastPCR software (Table 1). The extracted RNAs were reverse-transcripted into cDNA, followed by an amplification step, by using one-step RT-PCR kit (Qiagen, USA). The extracted RNA was reverse-transcripted in the presence of specific primer for 30 min and PCR reactions were run for 19–25 cycles of 94 °C (45 s), 63–64 °C (1 min) and 72 °C (45 s), using a thermal cycle (MJ Research, USA). Then, the 5 μL of PCR products were electrophoresized by 1.8% agarose gel and visualized under UV after ethidium bromide staining. The image captured using VersaDoc Imaging Device (BioRad, USA) and relative volume of bands was measured by Quantity One software (BioRad, USA).

### 4.10. Western Blotting Analysis

RAW 264.7 cells were induced with combination of 100 U/mL IFN-γ and 5 μg/mL LPS as described earlier and treated with different concentrations of BDMC33. To prepare the whole cell extract of RAW 264.7, the cells were scraped out of the culture flask and rinsed three times with ice-cold Tris-sucrose washing buffer. Then, the cell pellets was resuspended in lysis buffer (0.5% Triton-X, 2 mM EDTA, 2mM phenylmethylsulfonyl fluoride (PMSF), 2 ng/μL pepstatin A, 1 μg/mL leupeptin, 1 μg/mL apotinin, 10 mM Na_3_VO_4_, in Tris-HCl, pH 7.5) and incubated in ice for 30 min. Following incubation, the cells were sonicated at 20 Hz for 30 s. Then the cell was incubated for 20 min on ice and centrifuged at 25,150 *x g* at 4 °C for 30 min. The supernatants were collected and the protein content measured using a Bradford assay. In order to determine the expression of target protein, equal amounts of protein (10 μg) were electrophorized in a 10% SDS-PAGE and blotted onto a PVDF membrane (Amersham, UK). Membranes were blocked for 1 h at room temperature in a blocking buffer (5% non-fat dry milk, 0.081M Na_2_HPO_4_, 0.015 M NaHPO_4_, 0.15 M NaCl, pH 7.2, 0.1% Tween-20) and then incubated with primary antibodies for 1 h. The primary antibodies were used: rabbit polyclonal antibodies against iNOS (1:2000), ERK1/2 (1:2000), pERK1/2 (1:1000), p38 (1:2000), pp38 (1:1000), JNK1/2 (1:2000), pJNK1/2 (1:1000), i-κB (1:2000), pi-κB (1:1000) and HRP conjugated β-actin (1:2500). After several washes, HRP-conjugated donkey anti-rabbit IgG (1:1000–1:2000) were added and further incubated for 1 h. The proteins were detected by using ECL blotting reagent according to the manufacturer’s instruction. The image captured using VersaDoc Imaging Device (BioRad, USA) and intensity of bands was measured by Quantity One software.

### 4.11. Electrophoretic Mobility Shift Assay (EMSA)

RAW 264.7 cells were induced with a combination of 100 U/mL IFN-γ and 5 μg/mL LPS as described earlier and treated with different concentrations of BDMC33. The nuclear extracts were prepared by using the Nuc-Buster protein extraction kit (Novagen, Inc., USA) and EMSA or gel shift assay. This was performed using a LightShift Chemiluminescent EMSA Kit (Pierce, USA). Consensus sequence of NF-κB (5′-AGTTGAGGGGACTTTCCCAGGC-3′ and 5′-GCCTGGGAAAGTCCCCTCAACT-3′) and AP-1 (5′–CGCTTGATGACTCAGCCGGAA-3′ and 5′-TTCCGGCTG AGTCATCAAGCG-3′) were used for electrophoretic mobility shift assay after end labeling of the oligonucleotide with biotin using the Biotin Labeling Kit (Pierce, USA). Briefly, 30 μg of nuclear protein was incubated with binding buffer (10 mM Tris, 50 mM KCl, 1 mM DTT) supplement with 50 mM KCl and 50 ng/μL poly(dI.dC) for 10 min at room temperature before incubation with 50 fmol of labeled oligonucleotide for another 20 min at room temperature. The specificity of protein binding to the DNA was confirmed by competition reactions, in which a 120-fold molar excess of unlabelled oligonucleotide was incubated with the extracts simultaneously before the addition of the biotin-labeled oligonucleotide. DNA-protein complexes were analyzed by electrophoresis on 7% native polyacrylamide gel of TBE buffer (pH 8.5), followed by the transferring of DNA–protein complexes to a nylon membrane (Biodyne B Positively Charged Nylon Membrane). After transfer step, the membrane was immediately cross-linked at 120 mJ/cm^2^ using a commercial UV-light cross-link instrument (UV Stratalinker 1800) equipped with 254 nm bulbs for 1 min. A chemiluminescent detection method using a luminol/enhanced solution and a stable peroxide solution was used as described by the manufacturer, and the membranes were exposed in VersaDoc Imaging Device (BioRad, USA).

### 4.12. Fluorescence Microscopic Analysis of NF-κB Translocation

NF-*κ*B activation was assayed as translocation of the NF-*κ*B subunit p65 from cytosol to nucleus. RAW 264.7 cells were cultured on glass chamber slides and stimulated with IFN-γ/LPS with or without the presence of BDMC33. After treatment, the cells were washed with PBS, fixed with 0.2% glutaraldehyde plus 2% formaldehyde, permeabilized in 0.1% Triton X-100, and blocked in 2% bovine serum albumin for 1 h. Then, the cells were incubated with rabbit anti-p65 IgG (1:1000) for 1 h. After washing, the cells were incubated with FIT-C conjugated anti-rabbit IgG (1:1000) plus DRAQ5 (1:1000) for nuclei staining for further 1 h, and mounted with anti-fading reagent (Merck, Germany). The image visualized using Axiolab fluorescence microscope (Zeiss, Germany).

### 4.13. Flow Cytometry Analysis of TLR-4 and CD-14

A fluorescent-activated cell sorting (FACS) analysis was performed to detect the accessory molecules on the RAW 264.7 cells. The cells were seeded into 6-well plate at a density of 5 × 10^4^ cells, followed by stimulation and BDMC33 treatment as describe previously for 20 h. Cells were washed with washing buffer (0.2% FBS in PBS) and stained with Fluorescein isothiocyanate (FIT-C) conjugated anti-mouse TLR-4 and Peridinin Chlorophyll Protein Complex (PerCP) conjugated anti-mouse CD-14 at 4 °C for 30 min. The cells were washed twice by centrifugation in cold washing buffer at 350 g for 10 min each and acquired on a FACSCalibur (BD Biosciences, USA).

### 4.14. Statistical Analysis

Statistical analysis was performed using one–way analysis of variance (ANOVA) followed by Dunnett test *post hoc* for multi-group comparison test using GraphPad Prism software version 5.0. Statistical significance of differences between groups was accepted at *P* < 0.05.

## 5. Conclusion

**Figure 10:**
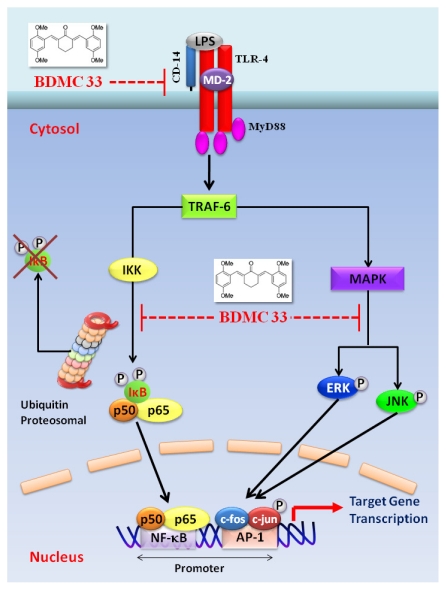
**A** schematic diagram shows the mechanisms underlying the inhibitory action of BDMC33. TLR-4 stimulation by LPS activates an intracellular signaling cascade that involves the recruitment of MyD88 (myeloid differentiation primary response gene-88) and results in the phosphorylation of TRAF-6 (tumor-necrosis-factor-receptor-associated factor 6), subsequently activate NF-κB and MAPK pathways. The red blunt lines indicate the inhibition by BDMC33.

In conclusion, we have demonstrated that BDMC33 effectively suppressed the production of NO, TNF-α and IL-1β upon IFN-γ/LPS induced macrophages. Moreover, the suppressive effects of the BDMC33 was associated with the down regulation of AP-1 DNA binding activity through blockade of the JNK and ERK signaling pathway; as well as inactivation of NF-κB signaling that resulted from the attenuation of the I-κB degradation, I-κB phosphorylation, NF-κB translocation and DNA binding activities, respectively (Figure 10). In addition, the suppressive effects of BDMC33 were partly mediated via reduction of CD-14 surface expression and not only restricted to the RAW 264.7 macrophages. Collectively, the experimental data suggests that the anti-inflammatory action of BDMC33 was attributed through interference in JNK-ERK-AP-1 and/or NF-κB signaling pathways in macrophages, which could be the possibility of its future pharmaceutical application in the control or management of inflammatory disorders.

## Figures and Tables

**Table 1:** The primer sequences of the target genes.

Target Gene	Gen Bank No.	Base Pair		Primer
TNF-α	**NM_013693**	324	Forward	5′-ACGGCATGGATCTCAAAGAC-3′
			Reverse	5′-CGGACTCCGCAAAGTCTAAG-3′
IL-1β	**NM_008361**	497	Forward	5′-TGACGTTCCCATTAGACAGC-3′
			Reverse	5′-TGGGGAAGGCATTAGAAACA-3′
GADPH	**M32599**	269	Forward	5′-TGTTCCTACCCCCAATGTGT-3′
			Reverse	5′-CCCTGTTGCTGTAGCCGTAT-3′
